# Community-based health professionals in face of crisis – a practice example from Bremen, Germany: Dirk Gansefort

**DOI:** 10.1093/eurpub/ckac130.001

**Published:** 2022-10-25

**Authors:** D Gansefort, T Altgeld, N Tempel, M Wächter, J Sterner, I Lettau, L Focke

**Affiliations:** Association for Health Promotion Lower Saxony, Hannover, Germany

## Abstract

At the end of 2020, a district-based analysis of Covid-19-infection rates in Bremen uncovered differences between socially deprived and “better off” areas. The first analysis conducted on a district level in Germany revealed a social gradient in the Covid-19-affectedness with deprived areas having above-average incidences. These areas are characterized by poor living conditions, high migrant population, low education and health literacy levels, language barriers and precarious job situations. Bremen's Health Senator acted on this challenge early on, initiated and financed the deployment of community-based health professionals (CHP) in disadvantaged areas to tackle the rising Covid-19-rates while reducing social inequalities and promoting health literacy. The CHPs’ work is coordinated by the Association for Health & Academy for Social Medicine Lower-Saxony. The 14 CHP are multilingual, with a background in health professions, social work and public health. They cooperate with local partners in 18 disadvantaged districts in Bremen and Bremerhaven. In 2021, work focused on outreach work, low-threshold provision of multilingual information (e.g., on regulations, vaccination) and the support of local vaccination campaigns. Bremen currently has the highest vaccination coverage in Germany (87,5% of population with primary series, national average: 76,2%; 07/2022).

Since 2022, CHPs’ work has shifted to health promotion activities aimed at mitigating the effects of the pandemic, e.g., physical inactivity, mental stress. Bremen was the first German city to pilot a large-scale implementation of CHP in socially disadvantaged areas. Due to their continuous presence, CHP quickly became people of trust and are integrated into local structures. Their work has received huge attention both in professional contexts and public media. The low-threshold outreach-work and close cooperation with local partners has high potential to address other health challenges in disadvantaged communities.

**Key messages:**

- Community-based health promotion with CHP is an effective way of reaching citizens in local settings and is able to adapt to unexpected social and health-related challenges.
- CHP strengthen the health literacy in the community and the communication of health regulations.

